# A versatile class of prototype dynamical systems for complex bifurcation cascades of limit cycles

**DOI:** 10.1038/srep12316

**Published:** 2015-07-22

**Authors:** Bulcsú Sándor, Claudius Gros

**Affiliations:** 1 Institute for Theoretical Physics, Goethe University Frankfurt, Frankfurt am Main, 60438, Germany

## Abstract

A general class of prototype dynamical systems is introduced, which allows to study the generation of complex bifurcation cascades of limit cycles, including bifurcations breaking spontaneously a symmetry of the system, period doubling and homoclinic bifurcations, and transitions to chaos induced by sequences of limit cycle bifurcations. The prototype systems are adaptive, with friction forces *f*(*V*(**x**)) being functionally dependent exclusively on the mechanical potential *V*(**x**), characterized in turn by a finite number of local minima. We discuss several low-dimensional systems, with friction forces *f*(*V*) which are linear, quadratic or cubic polynomials in the potential *V*. We point out that the zeros of *f*(*V*) regulate both the relative importance of energy uptake and dissipation respectively, serving at the same time as bifurcation parameters, hence allowing for an intuitive interpretation of the overall dynamical behavior. Starting from simple Hopf- and homoclinic bifurcations, complex sequences of limit cycle bifurcations are observed when the energy uptake gains progressively in importance.

The term ‘prototype dynamical system’ is employed for generic, but otherwise reduced systems, allowing to study and to understand a certain relevant phenomenon (like dynamical behavior and/or bifurcation scenario). For this, the dynamical behavior of the system should be dominated by the prime phenomenon of interest, with the system being otherwise simple enough to allow for straightforward numerical and (at least partial) analytic investigations^1^,^2^,^3^,^4^. Additionally, their dynamical behavior can often be understood in terms of general concepts, such as energy balance, symmetry breaking, etc.

Examples of prototype systems are the normal forms of standard bifurcation analysis^5^,^6^ and classical systems, like the Van der Pol oscillator^5^, or the Lorenz model^7^, which have been of central importance for the development of dynamical systems (systems) theory. As an example we consider the Liénard equation,


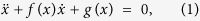


a generic adaptive mechanical system, which includes the Van der Pol oscillator and the Takens-Bogdanov system^8^,^9^. The periodically forced extended Liénard systems with a double-well potential have also been studied by many authors (see e.g. the double-well Duffing oscillator^10^,^11^,^12^).

In this paper we propose a new class of autonomous Liénard-type systems, which allow to study cascades of limit cycle bifurcations, using a bifurcation parameter controlling directly the balance between energy dissipation and uptake, and hence the underlying physical driving mechanism. Though there are a range of alternative construction methods for dynamical systems in the literature (see e.g.^13^,^14^,^15^), they generally involve abstract concepts, such as implicitly defined manifolds, or mathematical tools accessible only to researchers with an in-depth math training. In contrast to these methods, we provide here a mechanistic design procedure, based on the construction of attractors through the interaction of generalized friction forces with potential forces, an intuitive concept especially suitable for interdisciplinary investigations (e.g. in modeling cardiovascular systems^16^ or for solving optimization problems^17^), making it easily accessible and implementable for other scientific communities (such as neuroscience, biology etc.) as well.

As an introductory example for the role of balance between energy uptake and dissipation, in both local and global bifurcations, we reconsider the Bogdanov-Takens system,


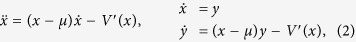


which is often used as a prototype system for homoclinic bifurcations^5^. Here, the mechanical potential is a third order polynomial, as illustrated in Fig. 1. The friction force is directly proportional to the velocity *y*, hence fixpoints of (2) correspond to the minima and the maxima of the potential *V*(**x**).

The dynamics of the Bogdanov-Takens system is controlled by the parameter *μ*, defining, in terms of the mechanical energy *E*, the regions of dissipation and energy uptake in the potential valley,


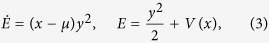


compare Fig. 1. The region *x* > *μ* of energy uptake increases when the bifurcation parameter *μ* is decreased, leading to two consecutive transitions. Initially the potential minimum becomes repelling, undergoing a supercritical Hopf bifurcation and a stable limit cycle emerges. Decreasing *μ* further, the extension of the limit cycle increases, merging at *μ*_*c*_ with the stable and unstable manifolds of the saddle, resulting in a homoclinic bifurcation.

## Results

The key mechanism leading to the bifurcations in the Bogdanov-Takens systems is the availability of a parameter, allowing to change the balance between energy uptake and energy dissipation along limit cycles. Our aim is to generalize this idea to the case of mechanical systems characterized by an arbitrary number of potential minima. For this purpose we consider


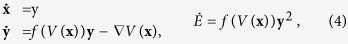


which describes a 2*d*—dimensional system, with *d* spatial coordinates, and friction forces *f*(*V*(**x**)) depending functionally only on the mechanical potential *V*(**x**), allowing for a fine-tuned control of the energy dissipation and uptake around the respective potential minima. A well known example of a system of type (4) is the Van der Pol oscillator:


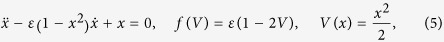


for which the regions of energy uptake and dissipation remain fixed, with *ε* regulating the overall influence of the velocity-dependent force.

The simplest generic class of friction functions *f*(*V*) entering (4) are polynomial:


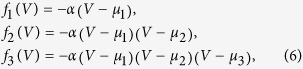


where *α* regulates the overall strength of the friction, and the individual *μ*_1_ < *μ*_2_ < *μ*_3_ are the respective zeros, the points at which dissipation changes to anti-dissipation and vice versa, compare Fig. 2.

**Figure 1:**
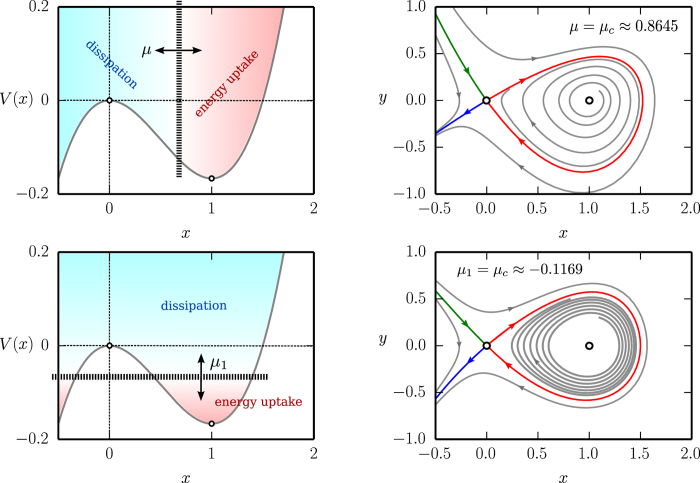
The Bogdanov-Takens system (2) with the potential function *V*(x) = *x*^3^/3 − *x*^2^/2 and a friction term *x* − *μ* (*top row*), and its generalization (4) to a friction term *μ*_1_ − *V*(x) (*bottom row*), compare Eq. (6). *Left column*: The potential function together with the color-coded regions of energy dissipation and uptake respectively, compare Eq. (3). *Right column*: The phase planes at the respective homoclinic bifurcation points, with the unstable foci and the saddles denoted by open circles. The green and blue trajectories are the stable and unstable manifolds, while the red trajectory corresponds to the homoclinic loop.

When using *f*_1_(*V*) and the mechanical potential *V*(**x**) = *x*^3^/3 − *x*^2^/2, the resulting flow in phase space is equivalent to the one of the Bogdanov-Takens system (2), as shown in Fig. 1.

### Generalized mechanical potentials

We are interested in using (4) as prototype dynamical systems, especially for the case of non-trivial mechanical potentials *V*(**x**) having an arbitrary number *M* of local minima. One could in principle consider higher-order polynomials for this purpose, however these do not allow to control the overall height of the potential and the relative width of the local minima in as simple a fashion.

For this purpose we use throughout this study potential functions of the kind


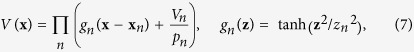


where the *z*_*n*_ > 0 determine the half-width of the respective local minima, and the *p*_*n*_ satisfy the self-consistent condition:


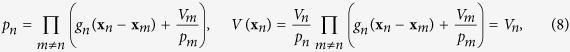


since *g*(0) = 0. For deep minima, with (*z*_*i*_ + *z*_*j*_) ≪ |**x**_*i*_ − **x**_*j*_|, the positions and the heights of the local minima are close to **x**_*n*_ and *V*_*n*_ respectively. We found that a relative accuracy of 10^−2^ for *V*_*n*_ can already be achieved in general after three or four iterations.

### Limit cycle bifurcation cascades

**Figure 2:**
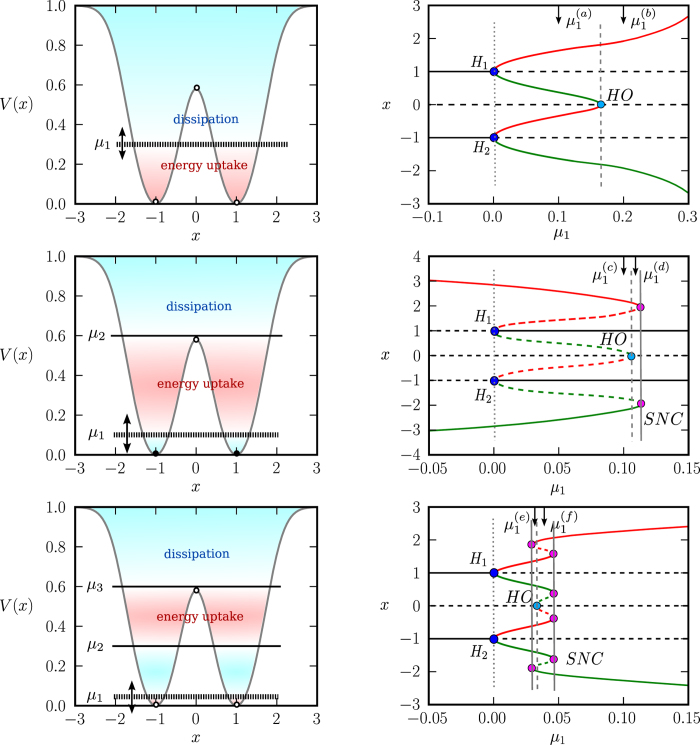
*Left column*: Double well potential, as defined by Eq. (7), with *x*_1,2_ = ±1, *V*_1,2_ = 0, *z*_1,2_ = 1 and *p*_1,2_ = 1.The regions of energy dissipation 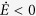 and uptake 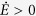 are color coded. For the friction functions (6) we used *f*_1_ with *α* = 1 (*top row*), *f*_2_ with *μ*_2_ = 0.6 and *α* = 5 (*middle row*), and *f*_3_ with *μ*_2_ = 0.3, *μ*_3_ = 0.6 and *α* = 5 (*bottom row*). *Right column*: Bifurcation diagrams of the respective generalized Liénard systems (4), functions of *μ*_1_. All other *μ*_*i*_ (when present) are kept constant. Stable/unstable fixpoints or limit cycles are denoted by continuous/dashed curves respectively. Black lines are fixpoint lines, while the maximal/minimal amplitude of *x* in a cycle is denoted with red/green color. *H* points denote Hopf bifurcations, *HO* corresponds to homoclinic bifurcations of a saddle, *SNC* points denote saddle node bifurcations of limit cycles. The dotted, dashed and continuous vertical gray lines are just guides for the eyes.

**Figure 3:**
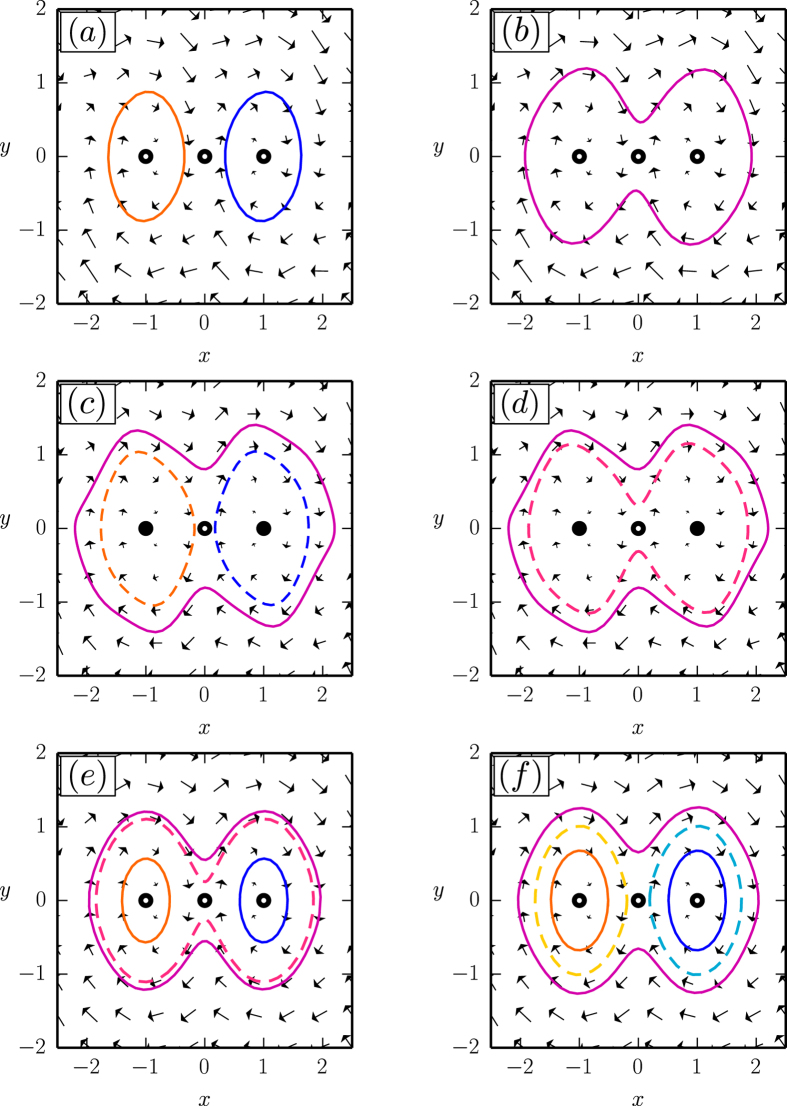
Flow diagrams for the systems presented in Fig. 2, using respectively linear/quadratic/cubic friction functions *f*_1_(*V*)/*f*_2_(*V*)/*f*_3_(*V*) (*top/middle/bottom row*). The values 
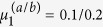
, 
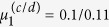
 and 
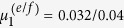
 for the respective *μ*_1_ are indicated by arrows in the corresponding bifurcation diagrams in Fig. 2.

The system of type (4) allows to describe complex cascades of limit cycle bifurcations. In Fig. 2 we show some illustrative examples, using a symmetric double-well potential and linear/quadratic/cubic friction functions *f*_1_(*V*)/*f*_2_(*V*)/*f*_3_(*V*) respectively, see Eq. (6). We used numerical methods (see the Methods section) to obtain the respective full bifurcation diagrams, with solid/dashed lines denoting stable/unstable fixpoints and limit cycles. The corresponding flow in phase space is illustrated in Fig. 3.

For negative *μ*_1_ the two fixpoints (±1, 0) are stable, for the case of *f*_1_(*V*) and *f*_3_(*V*), and stable limit cycles evolve via two supercritical Hopf bifurcations (bifurcations). For *f*_2_(*V*), on the other hand, a subcritical Hopf bifurcation is observed at *μ*_1_ = 0. The respective stable/unstable limit cycles merge for *f*_1_(*V*) and *f*_2_(*V*) in a homoclinic bifurcation, whereas a more complex bifurcation diagram emerges for *f*_3_(*V*). Saddle node bifurcations of limit cycles are present for both *f*_2_(*V*) and *f*_3_(*V*).

### Chaos via period doubling of limit cycles

**Figure 4:**
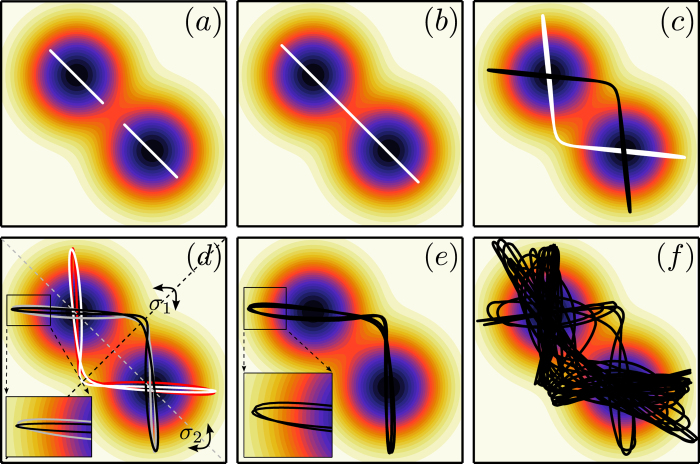
Stable limit cycles and chaotic orbits of the Liénard prototype system (4) in a two-dimensional symmetric double well potential *V*(**x**) (color coded, as defined by Eq. (9)) and with a linear friction term *f*_1_(*V*(**x**)) = 0.5 (*μ*_1_ − *V*(**x**)). The bifurcation parameter *μ*_1_ is 0.1, 0.15, 0.25, 0.265, 0.2698, 0.3 from (***a***) to (***f***). In (***d***) the four limit cycles can be mapped onto each other by using the symmetry operations *σ*_1,2_ or *σ*_3,4_, as discussed in the Methods section. For (***e***) only a single of the four stable limit cycles is shown. This needs to circle the two potential minima twice in order to retrace itself. In (***f***) an example of a chaotic trajectory is given.

**Figure 5:**
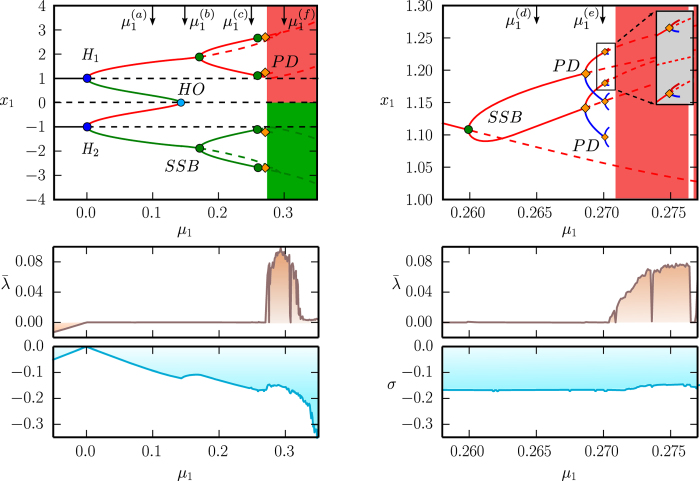
*Top row*: The numerically obtained bifurcation diagram for a four-dimensional prototype Liénard system with symmetric double-well potential and a linear friction force, as for Fig. 4. The second branches of limit cycles emerging from the two destabilized minima (see Supplementary Fig. S1) are not shown here. One observes Hopf and homoclinic bifurcations (H and HO), branching of limit cycles via spontaneous symmetry breaking and period doubling (SSB and PD), as well as a transition to chaos for *μ*_1_ > 0.2705. The red/green lines indicate the maximal/minimal *x*_1_-values of the respective limit cycles. The blue curve is the second *x*_1_-maxima after period doubling. The right diagram represents a zoom-in of the transition to a chaotic region, indicated by the shaded green and red areas. Only the first two period doubling bifurcations are shown. ***Bottom row*: The average Lyapunov exponent** 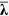 **and the contraction rate *σ*, calculated as described in the Methods section, for the corresponding *μ*_1_ parameter intervals**. For the left figure Δ*μ*_1_ = 0.001 parameter stepsize was used. Increasing the resolution more and more periodic windows (with 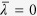) become visible, as shown on the right plot, where Δ*μ*_1_ is decreased by a factor of ten.

We consider now a prototype system (4) with a two-dimensional symmetric potential function *V*(**x**),


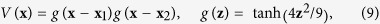


as defined in (7), having two minima **x**_1,2_ = ±(1, −1), and a linear friction term *f*_1_(*V*) = 0.5(*μ* − *V*). Both diagonals in the (*x*_1_, *x*_2_) plane are symmetry axes of the system, as discussed in the Methods section. In Fig. 4 we present examples of stable limit cycles and of a chaotic trajectory, as projected to the (*x*_1_, *x*_2_) plane. In Fig. 5 the corresponding bifurcation diagram is presented. The diagram shows Hopf bifurcations (H), homoclinic bifurcations (HO), branching of limit cycles via spontaneous symmetry breaking (SSB), period doubling of limit cycles (PD), and a transition to chaotic behavior:

**H** At 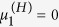 the two potential minima become unstable, just as for the one-dimensional spatial system presented in Fig. 2, resulting in two equivalent supercritical Hopf bifurcations. We note that, as a result of the symmetric potential function (9), a second branch of limit cycles is created by the two Hopf bifurcations (see the discussion in the Methods section and the Supplementary Information). However, since in the parameter region of interest these limit cycles are mostly unstable, we have not investigated them in detail.

**HO** At 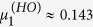 the limit cycles merge, as in Fig. 4(a,b), in a homoclinic transition. The limit cycle stays, however, exactly on the diagonal *x*_1_ + *x*_2_ = 0.

**SSB** At the first branching point of limit cycles, 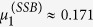 the symmetry with respect to the diagonal (1, −1) is spontaneously broken, as in Fig. 4(b,c), with the two limit cycles still being symmetric with respect to the (1, 1) diagonal. The latter symmetry is broken at the second branching point 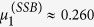, as in Fig. 4(c,d), creating four symmetry related stable limit cycles.

**PD** For larger values of the bifurcation parameter *μ*_1_ a series of period-doubling of limit cycles is observed, with the first occurring at 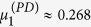, as in Fig. 4(d,e). The next period-doubling transition occurs at 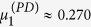, as shown in Fig. 5.

For reference we note that the saddle of the potential is located at *V*(0, 0) = 0.505, viz at a substantially larger value.

For 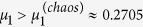 we observe seemingly chaotic trajectories, as illustrated in Fig. 4(f). Studying the transition to chaos is not the subject of the present investigation and we leave it to future work. We presume however, that the transition occurs via an accumulation of an infinite number of of period-doubling transitions of limit cycles, similar to the ones observed for the Lorenz system^18^ and for the Rössler attractor^19^,^20^.

Our prototype system (4) is not generically dissipative. We have evaluated the average contraction rate *σ*, as defined by (22) in the Methods section, and presented the results in Fig. 5. Phase space contracts trivially along the attracting limit cycles, but also, on average, in the chaotic region, where the average Lyapunov exponent 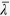 becomes positive. 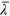 is negative for *μ*_1 _< 0, when only stable fixpoints are present, vanishing for intermediate values of *μ*_1_, when stable limit cycles are present. The later is due to the fact, see Fig. 7 and the corresponding Methods section, that two initially close trajectories will generally flow to the same limit cycle with the relative distance becoming constant.

**Figure 6:**
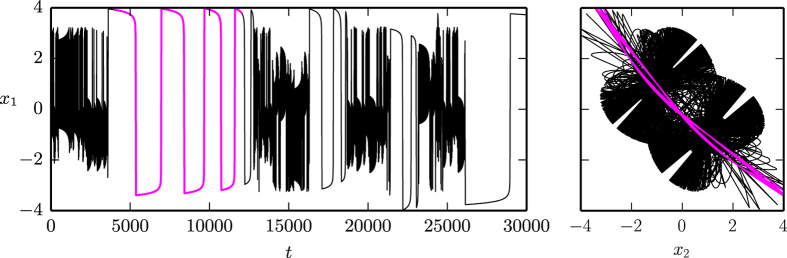
*Left*: Quasi-periodic windows with different time scales in the time-series plot of *x*_1_ for *μ*_1_ = 0.34. *Right*: Phase space plot of the trajectory projected to the (*x*_1_, *x*_2_) plane for the same parameter. The magenta curve corresponds to the quasi-periodic time interval *t*_*p*_ = [4 ⋅ 10^3^, 12 ⋅ 10^3^], denoted with the same color in the *x*_1_(*t*) plot.

For larger values of *μ*_1_ > 0.322 the chaotic region transforms into a phase of intermittent chaos as illustrated in Fig. 6, in which an extended quasi-regular flow along the (−1, 1) diagonal is interseeded by a roughly perpendicular bursting flow. This behavior is, to a certain extend, reminiscent to a scenario of intermittent chaos^21^, in which a strange attractor is embedded in a higher-dimensional space with partly unstable directions. We have, however, not investigated the observed intermittent dynamics in detail.

## Discussion

We have proposed and discussed a prototype dynamical system (4) in which the friction forces ∝ *f*(*V*) depend functionally only on the mechanical potential *V*(**x**). We have shown that complex cascades of limit cycle bifurcations can be obtained even for two dimensional phase spaces, when the friction function *f*(*V*) alternates between regions of energy uptake and dissipation.

We have also introduced a generic class of potential functions (7), which allows to define, in a relative straightforward manner, mechanical potentials with an arbitrary number of local minima and varying depth. Considering a simple double-well prototype system with two spatial dimensions (and with a four-dimensional phase space), we have shown that symmetry induced bifurcations of limit cycles and period-doubling of limit cycle transition to chaotic behavior can occur.

As discussed in the Methods section, the presence of stable and unstable fixpoints, the birth of limit cycles through transition from dissipation to energy uptake in the neighborhood of the local minima, or the symmetry properties of the prototype system do not depend on the particular method used to construct the potential function. The only requirement it has to fulfill is the existence of a certain number of local minima.

Hence, other potential functions could also be considered. For example, one could study the biquadratic version


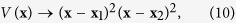


of the potential (9), used in our study of chaotic behavior with the prototype system (4). We did not study in detail the bifurcation diagram for the potential function (10), however, we have checked that one would get similar results to the ones presented in Figs 4 and 5, having the same underlying driving mechanism in terms of a linear friction function *f*(*V*) ∝ (*μ*_1_ − *V*). For increasing values of the *μ*_1_ control parameter, first stable fixpoints, then spatially separated-, merging- and symmetry breaking limit cycles can also be observed. Furthermore, using the potential function (10) chaotic behavior has also been found.

As a future perspective, we note that by changing the depth of the minima, one could control the order in which the fixpoints are going to be destabilized, which might lead to other interesting phenomena. Adding an extra (maybe slow) dynamics to the positions or depths of the minima, the metadynamics of the attractors^22^ may also be considered. In this case the *p*_*n*_ parameters should be recalculated in each time-step using the self-consistent equations (8), which proved to be fast enough for practical purposes.

Models, for which the equations of motion are derived from higher order principles, provide promising results for the understanding of many different phenomena, such as the optimization hardness of boolean satisfiability problems^17^ or the complex dynamics of biological neural networks^23^,^24^. Generally, these methods involve the construction of a generating functional, such as the cost function or energy functional^25^,^26^,^27^,^28^, with the dynamics of the system being defined by a gradient decent rule. When all equations are derived from the same generating functional, the system corresponds mathematically to a gradient system for which the asymptotic behavior is determined by stable fixpoints (nodes). They can thus not produce limit cycles or oscillatory behaviors. To by-pass this problem, additional equations of motions are usually defined, derived either from a second generating functional, to induce objective function stress^29^,^30^, or from other considerations. In our model, the system has an inherent inertia, which, in the presence of dissipation, leads to damped oscillations around the equilibria (minima of the potential). By creating regions of antidissipation, stable oscillatory dynamics and chaotic behavior is stabilized. Considering nonsymmetric and/or higher dimensional potential functions, we expect to find an even richer set of dynamical behaviors (see the Methods section), a scenario worth to be investigated in the future. As a possible application one could use the system for modeling the dynamics of various, complex and adaptive dynamical systems, for which the generalized potential function (energy landscape, cost functional, etc.) is approximated by (8) or is found from some other considerations. An alternative type of prototype dynamical system has been shown to be useful for understanding the coexistence of spiking and bursting neural activity observed in electrophysiological experiments^2^.

Finally, we note that the concepts of dynamical systems theory, such as attractors, slow points and bifurcations have been used recently to understand phenomena of surprisingly diverse fields. We mention here the modeling of birdsongs^31^ and migraine dynamics^32^, and the control mechanisms for the movements of humanoid robots^33^. The common approach, considered in these works, is the aim to construct simple and, to a certain extend, idealized dynamical systems, which allow for an in-depth understanding of certain dynamical behaviors. We hence believe that prototype systems allowing, in an intuitive manner, for the construction of models with a predefined set of attractors, as presented here, could offer a useful tool for understanding the behavior of a range of interesting interdisciplinary problems.

## Methods

The bifurcation diagrams shown in Figs 2 and 5 have been constructed by using the PyDSTool^34^ software package. In this section we provide the analytic calculations for the study of fixpoint stability for 2-, 3- and 2d-dimensional prototype systems respectively. We note that, these properties are valid irrespective of the particular shape considered for the potential function. This is followed by a discussion of symmetry properties, and presentation of numerical methods used to estimate the average Lyapunov exponent and the contraction rate.

### Hopf bifurcations in the prototype system

#### 2-dimensional prototype systems

The local maxima of the potential function, i.e. where 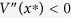, which are saddle points, separate the phase plane into different attraction domains with their stable manifold. Local minima with 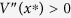 become, on the other hand, repelling focuses as a result of an Andronov-Hopf bifurcation, when dissipation changes to antidissipation in their neighborhood, having a simple pair of purely imaginary eigenvalues 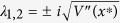.

#### 4-dimensional prototype systems

Analogously, the


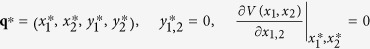


fixpoints of the 4-dimensional prototype systems (4) correspond to critical points of the *V*(*x*_1_, *x*_2_) potential function. Classification of the local minima and saddle critical points with respect to their stability can be achieved by evaluating the eigenvalues of the Jacobian of the system in terms of the Hessian of the potential function:


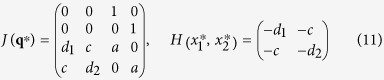


where we have defined the friction term and the second order partial derivatives of the potential at the respective critical points as


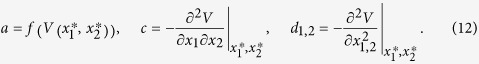


Defining also


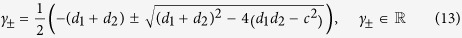


we can express the eigenvalues of the Jacobian *J*(q^*^) as


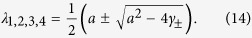


For general potential functions the local minima, defined by the conditions Δ = det(*H*) = *d*_1_*d*_2_ − *c*^2^ > 0 and *ρ* = tr(*H*) = −(*d*_1_ + *d*2) < 0 (or equivalently by *γ*_±_ > 0), undergo a Hopf bifurcation, when the *f*(*V*) friction term changes sign, i.e.:


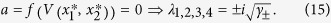


However, saddles of the potential function, i.e. Δ = det(*H*) < 0, are saddle type fixpoints of the dynamical system, having always a positive eigenvalue, as *γ*_+_ > 0 and *γ*_−_ < 0.

Here we note that in case of the potential function (9), due to the symmetries one gets a double pair of imaginary eigenvalues, since *d*_1_ = *d*_2_ and *c* = 0, and thus Eq. (13) yields *γ*_+_ = *γ*_−_. This results in a second branch of limit cycle solutions, not investigated in this paper, emerging from the Hopf-point.

#### 2d-dimensional prototype systems

For arbitrary dimensions *d* one can express the Jacobian in terms of block matrices:


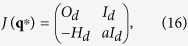


where *a* = *f*(*V*), and where *O*_*d*_ and *I*_*d*_ are the *d*-dimensional zero and identity matrices. 
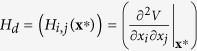
 is the Hessian matrix of the potential, evaluated for the respective q^*^ = (x^*^, y^*^) fixpoints.

To determine the eigenvalues of the Jacobian one has to solve the equation:


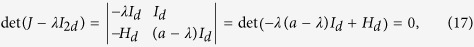


where we used the properties of square block matrices. By introducing *γ* = *λ*(*a* − *λ*) on finds with


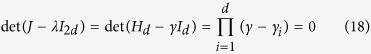


that the 2*d* eigenvalues *λ*_i_^±^ of the Jacobian can be expressed in terms of the *d* eigenvalues *γ*_*i*_ of the Hessian matrix and hence


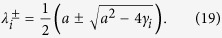


Consequently, at the local minima of the potential, i.e. when *γ*_*i*_ > 0, a Hopf-bifurcation occurs, with 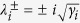, when the friction term *a* = *f*(*V*) changes sign. For general potential functions this might lead to the birth of higher dimensional tori or several branches of limit cycle bifurcations.

### Symmetries of the 4-dimensional system

The results shown in Figs 4,5 and 6 are found for the 4-dimensional prototype systems (4) with a linear friction force *f*_1_(*V*), as defined by (6), and a mechanical potential *V*(**x**) given by (9). The minima *V*(**x**_1,2_) = 0 of the potential, viz. **x**_1_ = (+1, −1) and **x**_2_ = (−1, +1) are connected by the symmetry operations


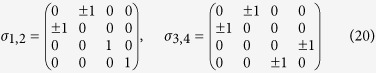


of the system. Thus, if (*x*_1_, *x*_2_, *y*_1_, *y*_2_) is a solution, then


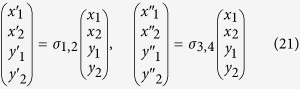


are also solutions.

As one could expect from the definition of the class of prototype systems introduced here (see Eq. (4)), the symmetry properties of the system are closely related to the particular symmetries of the potential function considered for modeling a certain behavior. Thus, finding the corresponding *σ*_*i*_ symmetry operations, could reveal new limit cycle solutions related by symmetry.

### Lyapunov exponent and contraction rate

**Figure 7:**
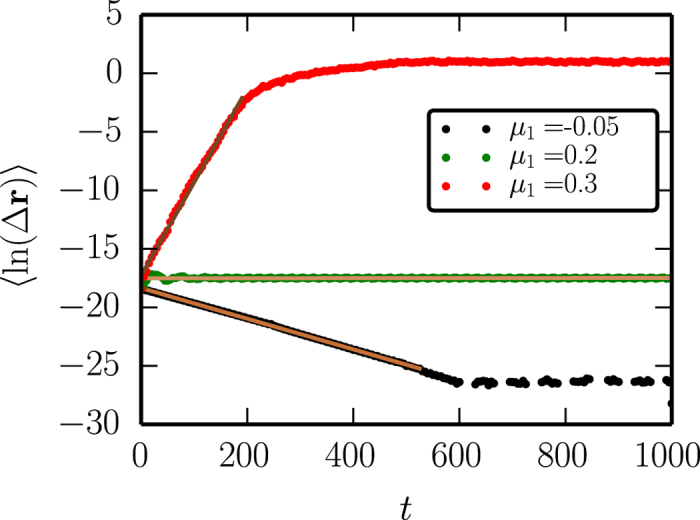
The logarithmic growth rate 〈ln(Δr)〉 averaged for 100 random initial conditions as a function of time for three qualitatively different types of dynamics: spiraling into a fixpoint (*μ*_1_ = −0.05), limit cycle oscillations (*μ*_1_ = 0.2) and chaotic behavior (*μ*_1_ = 0.3). Brown lines correspond to the best linear regression. In the first and last case, the line is fitted only to the first part of the trajectory. The dashed line indicates that the distance of the point pairs has reached the maximal accuracy of the integrator.

The local Lyapunov exponent *λ* is determined from the growth rate of the distance Δ**r**(*t*) = Δ**r**_0_*e*^*λt*^, between point pairs with an initial displacement, which we have taken to be Δ**r**_0_ = 10^−8^. The measurement of the Lyapunov exponent was started after a transient of *t*_*tr*_ = 1.5 ⋅ 10^4^. Considering 100 random initial conditions the average Lyapunov exponent 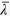 is then given by the slope of the initial linear part of the 〈ln(Δ**r**)〉 curve (as given by the brown lines in Fig. 7).

The contraction rate *σ*, is defined as the average of local contraction rates along a set of trajectories Γ for different initial conditions:


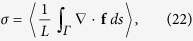


where *L*  =  ∫_Γ  _*ds* is the length of the trajectory, and **f** is the flow, viz the right-hand side of the evolution equations (4). *σ* is negative for dissipative systems, in which the phase space contracts^5^,^6^.

## Additional Information

**How to cite this article**: Sándor, B. and Gros, C. A versatile class of prototype dynamical systems for complex bifurcation cascades of limit cycles. *Sci. Rep.* **5**, 12316; doi: 10.1038/srep12316 (2015).

## Supplementary Material

Supplementary Information
