# Supplementary material for: A versatile class of prototype dynamical systems for complex bifurcation cascades of limit cycles
Source: Sci Rep. 2015 Jul 22;5:12316. doi: 10.1038/srep12316 (PMC4510578; doi:10.1038/srep12316)
Supplement: Supplementary Information [file srep12316-s1.pdf]

A versatile class of prototype dynamical systems for complex bifurcation cascades of limit cycles

Bulcsú Sándor and Claudius Gros*

Institute for Theoretical Physics, Goethe University Frankfurt, Germany

Supplementary note

In Supplementary Fig. S1 we present the second branch of limit cycles created by the Hopf bifurcations of the equilibria $\mathbf{q}^*_{\mathbf{1,2}} = (\pm 1, \mp 1, 0, 0)$, having a double pair of purely imaginary eigenvalues:

$$\lambda_{1,2,3,4} = \pm i\sqrt{\gamma}, \qquad \gamma = \left.\frac{\partial^2 V}{\partial x^2_{1,2}}\right|_{x_1^*, x_2^*}, \tag{S1}$$

as discussed in the Methods section. The limit cycles are unstable for almost the entire parameter region of interest, i.e. $\mu_1 \in [0, 0.35]$, except for the close vicinity of the Hopf point, where all the Floquet multipliers are just slightly smaller than 1.

Since these limit cycles have a perpendicular alignment in the (x_1, x_2) projection with respect to the limit cycles discussed in the main text of the paper, as shown in the right plot of Supplementary Fig. S1, they do not merge when the respective maximal and minimal amplitude curves cross in the bifurcation diagram (see the left plot of Supplementary Fig. S1). We note here that for $\mu_1 = 0.34$ the limit cycles already posses two stable manifolds, leading to the intermittent bursting seen in the dynamics found for this parameter, shown at the end of the Results section.

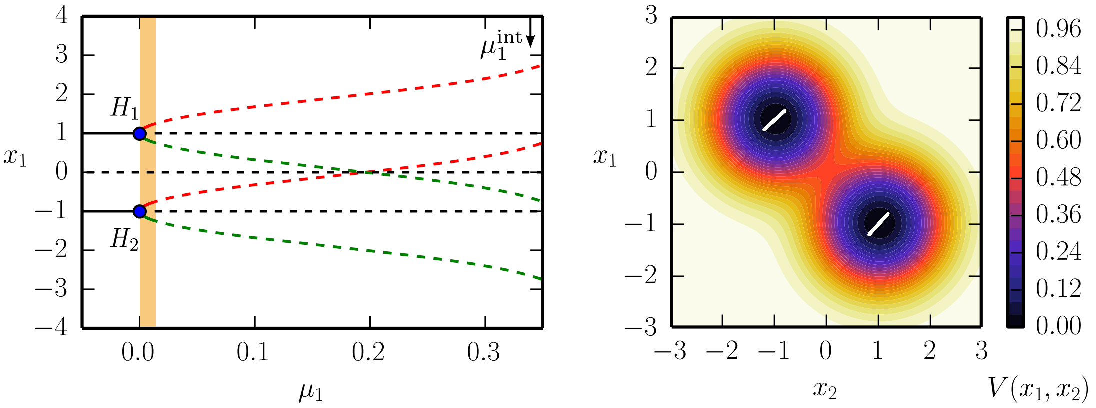

Supplementary Figure S1: *Left:* The numerically obtained second branch of limit cycles emerging from the two destabilized minima are shown here, together with the stable and unstable fixpoints, for a two dimensional prototype Liénard system with symmetric double-well potential and a linear friction force. Stable/unstable fixpoints are denoted by continuous/dashed lines. The red/green dashed lines indicate the maximal/minimal x_1-values of the respective limit cycles. The orange shaded area indicates the region in which the limit cycles are stable. The arrow in the top right corner shows the $\mu_1^{\rm int} = 0.34$ parameter value, for which the intermittent behavior shown in Fig. 7 was found. For the sake of clarity the limit cycles investigated in Fig. 5 are not plotted here. *Right:* The phase plane projection of the second branch of limit cycles for $\mu_1 = 0.008$, i.e. from the orange shaded region of bifurcation diagram shown in the left plot.

*Electronic address: gros@itp.uni-frankfurt.de
